# Defending against pathogens – immunological priming and its molecular basis in a sea anemone, cnidarian

**DOI:** 10.1038/srep17425

**Published:** 2015-12-02

**Authors:** Tanya Brown, Mauricio Rodriguez-Lanetty

**Affiliations:** 1 Department of Biological Sciences, Florida International University, Miami FL 33199

## Abstract

Cnidarians, in general, are long-lived organisms and hence may repeatedly encounter common pathogens during their lifespans. It remains unknown whether these early diverging animals possess some type of immunological reaction that strengthens the defense response upon repeated infections, such as that described in more evolutionary derived organisms. Here we show results that sea anemones that had previously encountered a pathogen under sub-lethal conditions had a higher survivorship during a subsequently lethal challenge than naïve anemones that encountered the pathogen for the first time. Anemones subjected to the lethal challenge two and four weeks after the sub-lethal exposure presented seven- and five-fold increases in survival, respectively, compared to the naïve anemones. However, anemones challenged six weeks after the sub-lethal exposure showed no increase in survivorship. We argue that this short-lasting priming of the defense response could be ecologically relevant if pathogen encounters are restricted to short seasons characterized by high stress. Furthermore, we discovered significant changes in proteomic profiles between naïve sea anemones and those primed after pathogen exposure suggesting a clear molecular signature associated with immunological priming in cnidarians. Our findings reveal that immunological priming may have evolved much earlier in the tree of life than previously thought.

The ability of the immune system to respond more rapidly and effectively to a pathogen that has been encountered previously is a trait well-characterized in vertebrates and mechanistically explained by the functional uniqueness of the adaptive immune system^1^. This trait has profound implications for a wide array of epidemiological and evolutionary phenomena. In contrast, it has long been assumed that invertebrates have an immune response that differs considerably from the acquired immune response found in vertebrates^1^.Invertebrates possess only an innate immune system, which is characterized by invariable pattern recognition receptors (PRRs) that target general pathogens-associated molecular patterns (PAMPs). The current consensus is that invertebrates lack the components of the adaptive immune system, such as those well characterized in vertebrates including highly variable major histocompatibility complex (MHC) receptors, immunoglobulins, and B and T cells that undergo clonal expansion and long term cell survival following antigen induced activation^1^,^2^,^3^,^4^. These elements of the adaptive immune system underlie the mechanism of the immunological memory phenomenon demonstrated in vertebrates. However, increasing evidence over the past years suggests that invertebrate immunity is much more complex than was generally believed. Immunological priming, the stimulation of the immune system with long-lasting effects that accelerate subsequent exposures to infectious pathogens, has been documented for a few groups of invertebrates, such as insects and crustaceans^5^,^6^,^7^,^8^,^9^,^10^. The first studies to demonstrate that initial pathogen exposure confers lasting specific protection were for the crustacean copepod *Macrocyclops albidus*^6^ and for the social insect *Bombus terrestris*^9^. Nevertheless, evidence of immunological priming in many other invertebrates remains absent, particularly for early diverging animals.

Cnidarians, including corals and sea anemones, are evolutionarily early-diverged metazoans and of great interest since some of these invertebrates can live for hundreds of years, suggesting they are potentially exposed to the same pathogens on many occasions during their lifespans. This has led to the mystery of how these long-lived organisms have done so well with only an innate immune system as the protective mechanism against infectious agents. As of yet, it is unknown if the defense response of these organisms strengthens upon repeated infections. Pioneering studies on coral skin grafts conducted in the late 1970s demonstrated that corals were able to reject skin grafts from genetically distinct donors more rapidly the second time the grafts were applied, suggesting a capacity for non-self recognition^11^,^12^,^13^. Self- and allogeneic recognition has also been described in sea anemones^14^ and soft corals^15^. While this phenomenon is not directly comparable with a defense against a pathogen, it might indicate that these basal metazoans have the capacity to remember foreign biological interactions. Additionally, understanding this aspect of immunology in corals and other cnidarians is imperative in light of the global concern of increasing epizootic disease outbreaks currently affecting the health of corals^16^,^17^,^18^,^19^ and the persistence of these fragile coral reef ecosystems^20^,^21^,^22^,^23^.

In the present study, we investigated whether priming is existent in cnidarians in response to pathogenic infections. If the protective response and survivorship of the host to pathogen challenges improve as a result of repetitive encounters with the infectious agent as compared to single encounters, it would suggest the existence of defense priming. Furthermore, presence of immune priming should also be associated with molecular changes underpinning the phenomenology, which is another fundamental investigation we conducted in this study.

## Results

### Host response to bacterial pathogen improves upon repeated pathogen encounter

**Figure 1:**
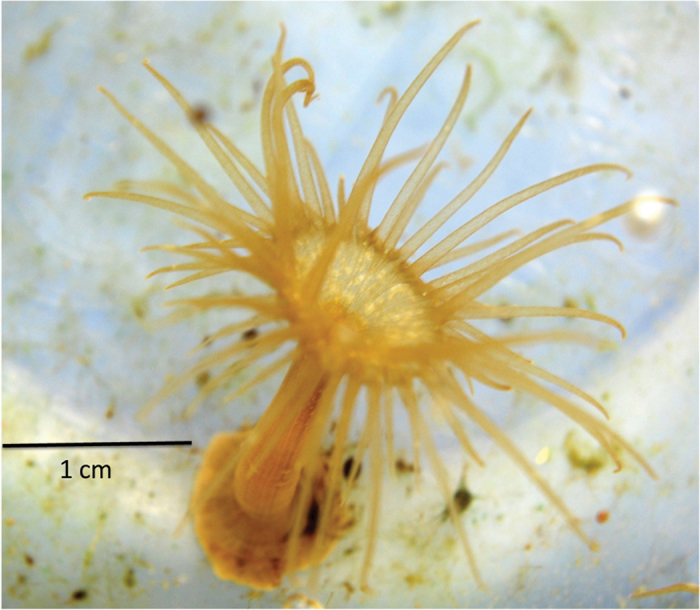
The sea anemone, *Exaiptasia pallida*, utilized in the immunological studies as a Cnidarian model system.

To test the hypothesis that a sub-lethal exposure of cnidarians to a pathogen induces a defense response that is memorized and expressed in an accelerated manner upon subsequent exposure, the host sea anemone *Exaiptasia pallida* (formerly *Aiptasia pallida*) was used as a cnidarian model (Fig. 1). *E. pallida* is easily reared and grown in the laboratory and these anemones closely resemble coral species in their associations with the same symbiotic dinoflagellate genus, *Symbiodinium*, and many of the same bacterial species^24^,^25^. These characteristics make the sea anemone an adequate system for asking biological questions of relevance for coral reef systems. The clonal line of sea anemones (CC7, originally obtained from Dr. John Pringle’s lab at Stanford University) was used for these experiments and has been reared in the laboratory for more than six years. Using the clonal line of anemones was advantageous as it removed any potential physiological and disease resistance variability that could be associated with unknown genetic differences among individual anemones found in a natural population. Moreover, the clonal anemones harbor the same symbiotic dinoflagellate type of *Symbiodinium* A4 (*sensu*: cp23S rRNA genotyping^26^), indicating that the photo-physiology of these clonal anemones is likely the same. Since experimental anemones were reared under the same environmental conditions for the last six years, the nutritional and physiological status of all anemones were presumed to be the same. The known coral bacterial pathogen *Vibrio coralliilyticus* was used as the infectious agent to elicit a defense response in the sea anemone. It is a major coral pathogen known to cause coral bleaching^27^ and white syndrome in *Acropora* corals^28^. It has also been shown to cause disease and mortality in *Exaiptasia pallida*^29^. The anemone responds to *V. coralliilyticus* with darkening of the tissues and retraction of tentacles, followed by complete disintegration of polyp tissues^29^,^30^. The disease progression pattern is consistent with the behavior of necrotizing pathogens^29^.

To assess the response of *E. pallida* anemones to repetitive encounters with the infectious agent, we first determined a sub-lethal exposure of the bacterial pathogen *V. coralliilyticus* that would allow priming of the host without causing mortality. It was determined that a concentration of 1 × 10^8 ^CFU ml^−1^ of this bacterial agent causes stress and mortality in *E. pallida*anemones after four days of exposure (Supplementary Fig. S1A online). Within a ten-day bacterial exposure, mortality ranged from 60 to 90% in the anemones (Supplementary Fig. S1A online). However, if anemones were removed from the bacterial challenge, washed, and placed in pathogen-free seawater after the third day of pathogen exposure, anemones would recover and show 100% survivorship comparable to unexposed (control) anemones (Supplementary Fig. S2 online). Based on these results, a sub-lethal challenge of a three-day pathogen exposure at 1 × 10^8^ CFU ml^−1^ was used for the immune priming experiments. The bacterial challenges were conducted at 30 °C as it has been shown the virulence in this pathogen increases at temperatures above 28 °C^27^,^31^. We demonstrated that this experimental temperature was not a factor of mortality during the bacterial exposure trials (Supplementary Fig. S1B online).

**Figure 2:**
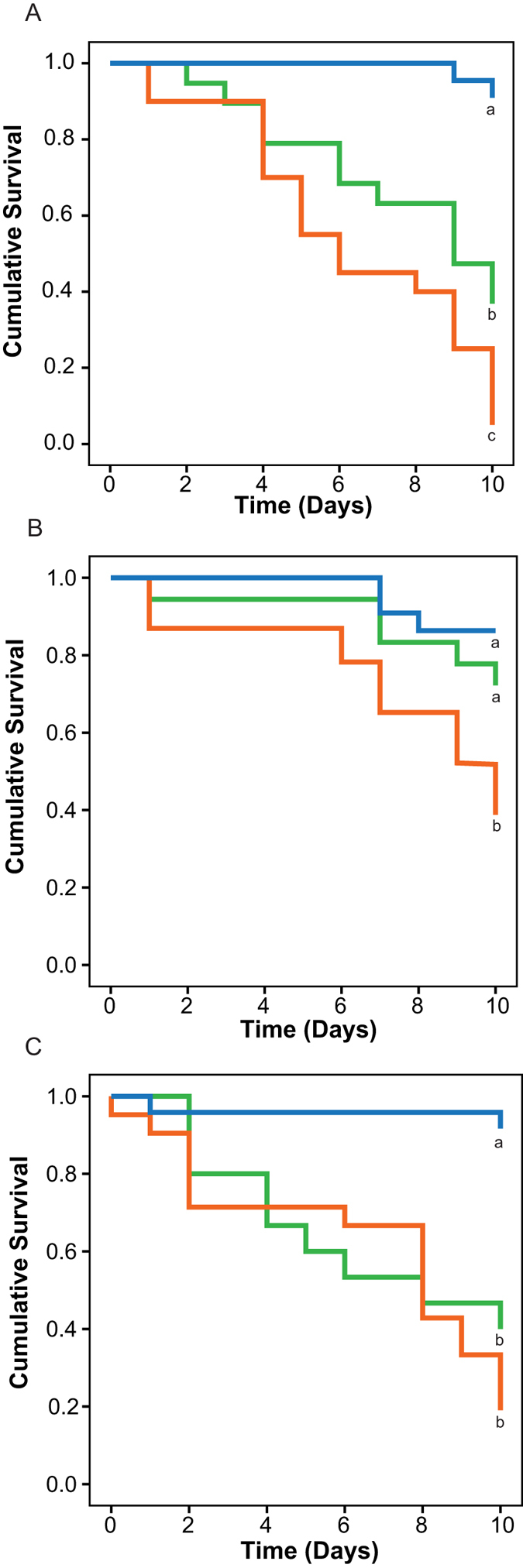
Kaplan-Meier survival plots for *Exaiptasia pallida* during 10-day lethal challenge to the pathogen *Vibrio coralliilyticus* following: (A) two weeks; (B) four weeks; and (C) six weeks recovery period post priming with a sub-lethal exposure. Green lines indicate those anemones that were primed with a sub-lethal exposure prior to the lethal challenge; orange lines represent anemones that were exposed only to the 10-day lethal challenge with no prior priming, and blue lines indicate control anemones that were not exposed at all to the pathogen. Different letters next to the graphed lines indicate statistically significant difference among the treatment at p < 0.05 (Kaplan-Meier; Mantel-Cox Post hoc test) N = 20 per treatment.

Following these trials, three experiments were performed to evaluate the existence of a priming response. Anemones were first subjected to a sub-lethal exposure of *V. coralliilyticus* followed by resting periods (pathogen-free recovering time from the sub-lethal challenge) of either two, four, or six weeks before exposing the sea anemones again to a lethal exposure (ten day pathogen challenge). It is important to note that none of the anemones died during the resting period or prior to the lethal challenge. The response and survivorship of these anemones (primed group) were compared to anemones that were exposed to a lethal challenge but without prior sub-lethal exposure (non-primed group), and also to a control group in which anemones were never exposed to sub-lethal or lethal bacterial challenges. The results showed that anemones that had previously encountered the pathogen (primed) had a higher survivorship than those anemones that encountered the pathogen for the first time (Fig. 2; Kaplan Meier; Mantel – Cox Post hoc test, p = 0.0001). The survivorship rate appeared to vary as a function of the lapsed time between the two consecutive pathogen exposures. Anemones exposed to the lethal challenge two and four weeks after the sub-lethal exposure presented seven- and five fold increases in survival, respectively, compared to the non-primed anemones (Fig. 2A-B; Kaplan-Meier; Mantel - Cox Post hoc test; Two weeks, p = 0.031; Four weeks, p = 0.039). However, the experimental group of anemones challenged six weeks after the sub-lethal exposure showed a 1.4 fold increase in survivorship that was not statistically significant (Fig. 2C; Kaplan-Meier; Mantel - Cox Post hoc test, p > 0.05). The improved response of the anemones to repeated encounters of the pathogen suggests the existence of transient defense priming that lasts for up to four weeks.

To determine whether the improved response in the primed anemones was not due to a chronic infection that continued over the experimental period, the pathogen load on the subjected anemones after the sub-lethal exposure was quantified using quantitative PCR (qPCR). Results from the assay indicated that by day four of the recovery period, *V. coralliilyticus* was no longer detectable in *E. pallida* (Supplementary Fig. S3A online; ANOVA, p < 0.05), suggesting that the improved response to pathogen upon repetitive encounters was due to a priming phenomenon and not to chronic infection. The same qPCR assay was used to confirm that *V. coralliilyticus* was present in *E. pallida* hosts throughout the lethal experiment, which showed considerable presence of pathogen load throughout the lethal exposure (Supplementary Fig. S3B online).

### Proteomic analysis to dissect the molecular changes associated with the immune priming response

**Figure 3:**
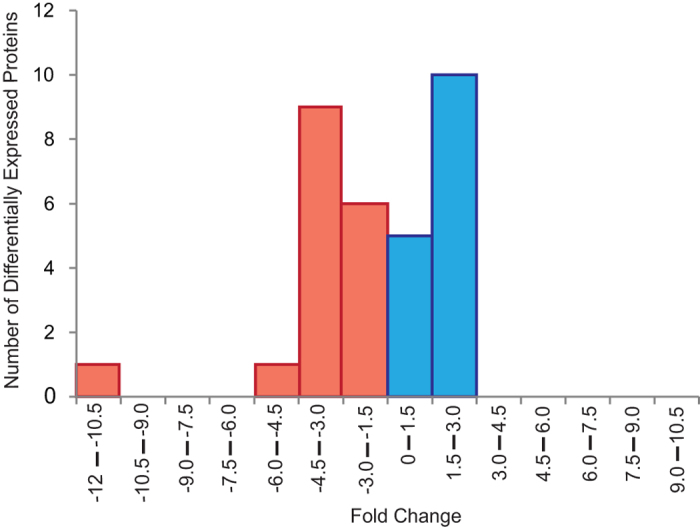
Histogram of the differentially expressed proteins as a function of fold change from *Exaiptasia pallida* anemones four weeks post priming in comparison to naïve anemones never exposed to the pathogen. Blue bars indicate proteins that were differentially up-regulated in the anemones previously primed with a sub-lethal exposure to the pathogen; and red bars indicates down-regulated proteins in the same treatment.

**Figure 4:**
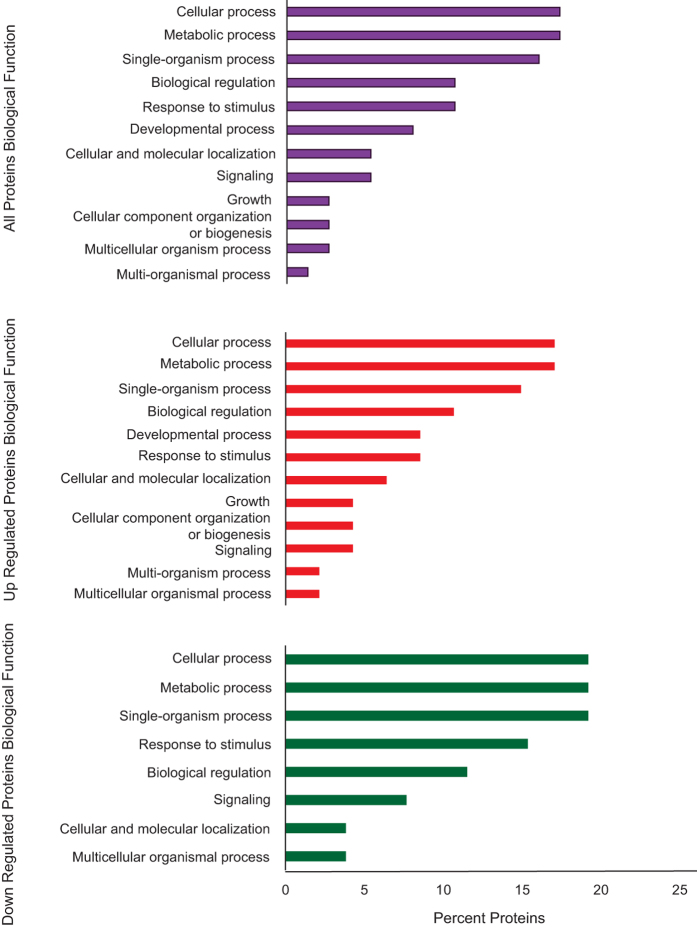
Representation of biological processes Gene Ontology (GO) terms for the 32 *Exaiptasia pallida* characterized proteins. The results are summarized in three subgroups: all GO terms, GO terms from up-regulated and down-regulated proteins.

Further exploration of the immunological priming phenomenon took place at the protein level by analyzing samples collected four weeks following the post-priming phase and immediately before the lethal exposure. A two-dimensional fluorescence gel electrophoresis combined with mass spectrometry was used for the analysis. Based on the replicated 2D fluorescent in-gel analysis, a total of 1400 spots were detected. From this proteome, 39 spots (2.79%) with at least 1.3-fold change were identified as being differentially expressed between primed and non-primed anemones four weeks after the sub-lethal exposure to the primed anemones (Biological Variation Analysis, BVA, p < 0.05). Among these proteins, 16 were up-regulated, and 23 proteins were down-regulated in the primed anemones (Fig. 3). Of the 39 identified spots, the protein identities of 32 spots were determined using MALDI-TOF mass spectrometry and proteomic database comparisons with high confidence (Confidence Interval >95%; table 1). The protein profiles showed surprising complexity as some of these spots represented multiple isoforms of proteins varying in molecular mass and/or charge (Supplementary Fig. S4 online). The differentially expressed proteins identified through this method were involved in 27 different biological processes. The most represented biological processes were metabolic process (n = 14, GO: 0008152), cellular process (n = 13, GO: 0009987), response to stimulus (n = 8, GO: 0050896), and single organism process (n = 12, GO: 0044699) (Fig. 4, Supplementary Table S1 online). The representation of biological processes was similar between up-regulated and down-regulated proteins except the categories of developmental processes and cellular component organization and biogenesis, which were only represented by up-regulated proteins.

Of the proteins identified in this study, heat shock protein 70 was the most highly up-regulated protein in primed *E. pallida* with a 2.02-fold-higher expression in primed as opposed to control anemones (Table 1). Interestingly, a second protein also identified as heat shock protein 70, based on amino acid sequence of the generated mass-spec polypeptides was the most highly down-regulated protein with a 9.73 fold decrease in expression in primed anemones when compared to controls. However the size of this protein on the gels does not correspond to a 70 kda protein (Supplementary Fig. S4 online) indicating that this protein might be a smaller Heat Shock Protein.

## Discussion

The sea anemone, *Exaiptasia pallida*, showed susceptibility to bacterial challenge with *Vibrio coralliilyticus* similar to coral species affected by the same bacterial pathogen at seawater temperatures 3 to 5 °C warmer than ambient temperatures^27^,^31^. However, the survivorship of the challenged anemones depended on previous pathogen exposure. Anemones that encountered the pathogen in sub-lethal conditions prior to lethal exposures showed higher survivorship than naïve anemones encountering the pathogen for the first time. Such findings suggest the potential presence of a protective priming defense mechanism in some members of the phylum Cnidaria. The priming response was also shown to be short-lived, lasting up to one month. The improved response of primed compared to non-primed anemones was not the result of a sustained immune response due to a chronic infection from the sub-lethal exposure, as the pathogen *V. corallilyticus* was cleared by the sea anemones four days after the termination of the sub-lethal exposure and several weeks before the second pathogen exposure. This is critical as a chronic infection in which a low level response of the immune system continues actively combating an infection^32^ and cause a more rapid secondary response in a subsequent pathogen challenge due to an already engaged immune system of the attacked host^9^. It is important to note that it is possible that even though *V. corallilyticus* presence decreases beyond detection, the presence of large concentrations of *Vibrio* at the onset of the experiment may have impacted the other microbial species associated with the anemone that may function as beneficial symbionts. Further studies are required to explore this possibility. Our findings demonstrate that the cnidarian defense system is functionally capable of unexpectedly durable induced protection. This suggests that selective pressures that triggered the evolution of immunological priming have a signature from early diverging animals.

Immunological priming has been documented in other invertebrates including crustaceans and insects^5^,^6^,^7^,^8^,^9^,^10^. In these cases, the improved response to the pathogens upon multiple encounters was also shown to be short-lived. For instance, the social bumble bee, *Bombus terrestris*, gain increased protection against pathogens upon a secondary exposure that lasted up to 27 days^9^. In this case, the priming of this duration would compare with an average life span of around 4 weeks for adult *B. terrestris* workers in the field. It highlights the clear ecological, and thus evolutionary, benefits of immunological priming in these organisms. In the case of cnidarians, such as anthozoans that can live for hundreds of years, immunological priming that confers a lasting protection of a month might appear to have a low ecological and evolutionary value. Interestingly, a similar timeframe of priming has also been described within the context of allorecognition in cnidarians. For example, specific memory of tissue transplantation immunity has been demonstrated to last four weeks in the coral *Montipora verrucosa*^13^, and eight weeks in the gorgonian, *Swiftia exerta*^15^. Yet some corals, such as the hydrocoral *Millepora dichotoma* appear not to possess a memory component to allorecognition^33^. The three above examples show a varying memory response to previously encountered allografts. In the cases that do show allomemory, it appears also to be relatively short-lived. While the mechanisms used in alloimmunity by cnidarians are unknown, it is possible that common ground might exist between the mechanisms modulating the processes of allografting and pathogen recognition. Regardless of the similarity between the mechanisms, the question remains as to what would be the advantage for a long-lived organism to have such a short-lived priming of their defense system. We speculate that short-lasting priming of the defense response could be ecologically relevant if pathogen encounters are concentrated and restricted to particular seasons (short period of time) characterized by high stress. In such seasons when pathogens are more active and virulent, cnidarians could be ecologically and evolutionary benefited if they have the capability to remember pathogenic encounters during the duration of the high stress season. This would allow maximizing the allocation of energy towards immunological priming when it is most needed since it has been shown to be energetically taxing on other organismal processes^34^. In recent years we have learned that sustained high temperatures above the average seasonal maximum are often related to an increase of disease outbreaks^21^,^23^,^27^,^35^,^36^,^37^. For example, Bruno and collaborators (2007) showed a highly significant relationship between the frequencies of warm temperature anomalies and the occurrence of white syndrome in Pacific reef-building corals. High temperatures during the summer months have also been associated with the activation of virulence among bacterial pathogens that are common residents in the coral reef environment^27^,^38^,^39^. Moreover, the high virulence and disease prevalence is seasonal in the majority of cases and ceases as the water temperature declines at the end of the summer^17^,^21^. Therefore, it is conceivable that the short duration of immunological priming described for *Exaiptasia pallida* mirrors the short time window when pathogens are seasonally active and during which they could be encountered repetitively by the sea anemone. This strategy would allow the sea anemone to reallocate energy use to other vital physiological needs during times where pathogens are less infectious. Additionally, the duration of priming might also be pathogen-specific. For example, in mice different pathogens elicit varying durations of priming and memory^40^. Pathogens that are encountered more often by a given organism could cause longer priming/memory. Further investigations are needed to understand whether different pathogens trigger longer immunological priming, and also to reveal how pathogen-specific the priming phenomenon is in the defense system of cnidarians, which could shed light about the existence of immunological memory.

### Proteomic analysis of immune priming in *E. pallida*

The phenomenological data presented above indicate that there are responses in cnidarians that might be trained by past experience and increase upon a second exposure. This adds to the growing notion that invertebrates have extremely plastic immune effectors that can generate novel and functional immune response changes in relation to past experience. In the past the logical fallacy that because an organism lacks B and T cells, the organism will also lack an adaptive immune response has hindered our appreciation of the capability of basal metazoans to possess immunological priming and memory^41^. These organisms could generate a trained immune response in another way as has been documented for insects^42^,^43^. In our study we attempted to characterize molecular changes correlated with the priming phenomenon using a comparative proteomic approach to start dissecting the mechanism underlying an inducible enhanced immunity in cnidarians.

Statistically significant differences in proteomic profiles between naïve sea anemones and those primed after pathogen exposure suggests a clear molecular signature associated with immunological priming in cnidarians. The group of differentially expressed genes was diverse, suggesting that the molecular regulation of the priming defense is governed by changes in multiple cellular processes. None of these proteins were identified as antimicrobial peptides, which are the molecules normally produced during the actual fighting and clearing of pathogen infections in cnidarians^44^,^45^,^46^. In other invertebrates such as bumblebees, antimicrobial peptides are produced immediately after the bacterial challenge and then subside thereafter when the pathogen load decreases^9^. Therefore, the lack of differentially expressed antimicrobial peptides was expected as the proteomic analysis was conducted in primed anemones at least three weeks after the pathogen from the sub-lethal exposure was cleared. Consequently, the changes in protein expression detected in the primed anemones four weeks after the first pathogen exposure seem to be related the phenomenon of immunological priming rather than to pathogen clearance.

Gene ontology analysis indicated that many of the differentially produced proteins linked to immune defense priming were grouped into metabolic processes pathways, a pattern that has also been detected from transcriptomic analyses in corals affected with disease signs^47^,^48^. An example of a metabolic protein is the Fructose-Bisphosphate Aldolase protein that is an enzyme involved in glycolysis, a metabolic process that assures the production of energy required for a large number of other metabolic processes. We detected a higher amount of this protein in primed anemones, suggesting an enhanced metabolic function in primed animals. Previous studies have also shown a correlation between increased expression of metabolic enzymes in the response to secondary exposure of pathogens^49^. Recently, it was proposed that a shift of central glucose metabolism from oxidative phosphorylation to aerobic glycolysis (the “Warburg effect”) is the metabolic basis for trained immunity (i.e. the memory characteristics of the innate immune system recently described in vertebrates^50^), providing the energy and metabolic substrates for the increased activation of trained immune cells^51^. Further experimental and physiological studies are needed to investigate whether an increase of glycolysis is indeed a fundamental process in primed “trained” immunity in early-diverging metazoans, such as cnidarians. Findings from these future research avenues will provide an appreciation of the evolutionary origin for the key role of metabolism in innate host defense.

Many of the identified proteins in this study show homology to immune genes functionally characterized in other organisms. Although we need to be cautious when borrowing functionality of these proteins based on homology to other organisms^52^, their expression in this study bolsters the idea that they may be involved in the immune response of *E. pallida*. In the context of cnidarian immunology, we discuss key changes in protein production involved in following functional groups: stress response, ion transport and proteolysis.

**Figure 5:**
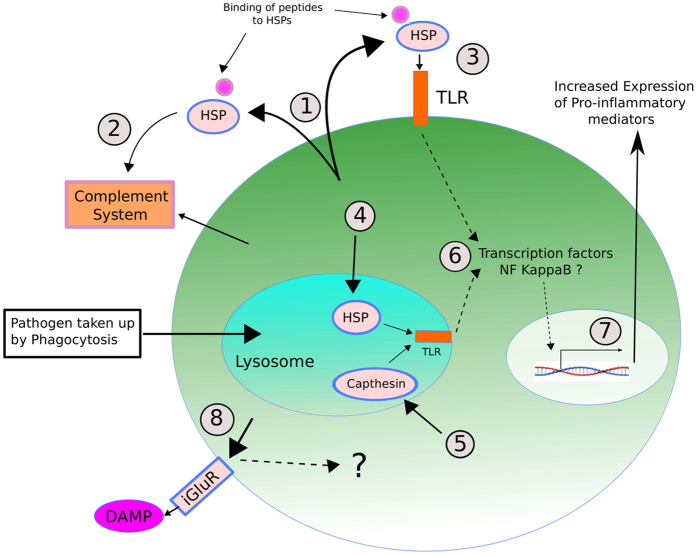
Proposed model of Heat Shock Proteins (HSP),Capthesin and Glutamate Receptor (iGluR) roles in cnidarian molecular defense priming: (1) HSP are up regulated and some are extracellularly secreted where bind to peptides and act as DAMPs; (2) as DAMPs, HSP help with a faster activation of the innate complement system, and/or (3) interact and cause a quicker activation of outer host cell membrane TLRs; (4) intracellularly, up-regulated HSP proteins can be delivered into lysosomes in which they can also interact and activate endosomal cell membrane TLRs; (5) higher production of Capthesin are delivered into lysosomes in which they can also interact and activate endosomal cell membrane TLRs; (6) activated TLRs either from the outer membrane or endosomal membranes will trigger cell signaling pathways that will converge in the activation of transcription factors (likely NF-kappa β) that will ultimately induce the expression of immune-related genes (7) resulting in the production of potential pro-inflammatory molecules; (8) Higher expression of iGluR expressed on the outer membrane will also facilitate a faster sensing of potential DAMPs upon secondary exposure of pathogens.

Several proteins involved in stress response were detected in association with defense priming: two putative heat shock protein 70 (HSP70) orthologs and one heat shock protein 60 (HSP60) were up-regulated whereas three small heat shock proteins (~20 kDa) were down-regulated. It is well-known that the up-regulation of HSP synthesis provides resistance to toxic stresses such as heat shock^53^,^54^; however, their involvement has also been shown in response to many other environmental and biological insults, such as pathogenic infections^55^. Recently, we also documented transcriptional up-regulation of a HSP70 gene in the scleractinian coral, *Acropora millepora*, within hours of exposure to bacterial pathogens^56^. There is still no clear understanding of the molecular mechanisms involving HSP in response to pathogenic infection and whether its action in the immune defense is at the intracellular and/or at the extracellular level. However, it has been suggested that many HSPs have the property of damage associated molecular patterns (DAMPs) as they can bind to exposed hydrophobic residues of a wide spectrum of polypeptides^57^. HSPs could play a critical role in mediating innate immunity by activating Toll-like receptor (TLR) signaling due to their status as DAMPs and thus induce cytokine-mediated inflammatory responses. For instance, some evidence indicates that extracellular HSP70 can interact with TLR4 under a number of pathological situations^58^,^59^. Furthermore, HSP70 has been implicated in immunity stimulation either by antibody-independent activation of the complement immune system^60^ or by enhancing the expression of the prophenoloxidase system^61^. Under the hypothesis of DAMP-acting HSPs, it is possible that a higher synthesis of HSPs in primed organism, such as in the case of the sea anemone from this study, could allow for a faster response at the detection of infection-associated danger through interaction of TLR-HSP-DAMP, and thus induce a quicker inflammatory response upon a new exposure to pathogens (see model at Fig. 5). This model is supported by studies conducted on the brine shrimp *Artemia* showing that heat-induced accumulation of HSP70 appears to protect crustacean from pathogenic infection by *Vibrio campbellii*^62^. Current findings have shown that direct delivery of HSP vaccines to crustaceans improve their defense response and success to fight infections of pathogens^61^,^62^,^63^. For example, feeding with *E. coli* YS2 over-producing DnaK, the prokaryotic equivalent of Hsp70, enhances gnotobiotic *Artemia* larvae survival approximately two- to three-fold upon challenge with pathogenic *V. campbellii*^63^.

Other proteins detected in this study were putatively characterized as being involved in ion transport. One of particular attention was the inotropic glutamate receptor (iGluR)-like protein found to be up-regulated in the primed anemones. iGluRs are ligand-gated ion channels best known for their role in fast excitatory neurotransmission in vertebrate and invertebrate nervous systems. However, new findings have shown that many homologs of these receptors are implicated in other biological process. A novel family of iGluR-related genes from insects (referred to as Ionotropic Receptors, IR) have been characterized as chemosensory receptors and are involved in olfaction and gustation processes^64^,^65^. The findings that these receptors are present across diverse groups of organisms from bacteria, plants and animals also suggests that this receptor family represents an evolutionarily ancient mechanism for sensing both internal and external chemical cues^64^. Of great interest are the findings showing that plant iGluRs are implicated in sensing a broad range of amino acids as part of the defense mechanism against infectious agents^66^. Recent studies examining the wound response and disease susceptibility in *Arabidopsis thaliana* Glutamate-Like Receptors (GLR) knockout mutants have provided evidence that some members of the GLR gene family encode important components of the plant’s defense response^67^. These discoveries are in line with our finding of higher production of iGluR-like proteins in primed *Exaiptasia* anemones. If involved in recognizing/sensing danger associated molecular components, a primed anemone with higher levels of expression of iGluRs would be better prepared to respond faster to infectious agents upon secondary exposures (Fig. 5).

Finally, we detected a higher production of immune-related proteolytic proteins including aminopeptidases and cathepsin, suggesting a potential enhancing key role of proteolysis in immune priming. For instance, capthepsins are key lytic enzymes and members of the proteases machinery packed in host lysosomes. These enzymes are not only involved in lysosome-contained pathogen degradation but also have been implicated in activating endosomal Toll-Like Receptors (TLR), which induce downstream cytokine-mediated pro-inflammatory responses^68^,^69^. Cathepsins generate a proteolytic cleavage, a prerequisite for TLR7 and TLR9 signaling^70^,^71^. Greater amounts of this protein in primed anemones implies that up-take of pathogens via phagocytosis will be digested and cleared faster through the phagosome-lysosome pathway. Additionally, their recognition by TLRs could be enhanced by a higher rate of proteolytic cleavage.

In summary, while immune priming has been found in several invertebrates^6^,^9^, this study discovered for the first time a similar phenomenon in an early diverging animal. Our findings support the notion that immunological priming may have evolved much earlier in the tree of life than previously thought. Additionally, the considerable amount of proteins that appear to be involved in the immune response of primed *E. pallida* suggests immunological priming in cnidarians is a more complex phenomenon than so far has been recognized. Furthermore, finding immunological priming in a sea anemone implicates the potential presence of the same mechanisms in other cnidarians such as corals. Future research addressing these mechanisms might be of crucial influence on developing restoration strategies for threatened and endangered coral reef species. As a potential outcome, “immunization” could become a tool to improve tolerance and survivorship of long-lived wild and re-introduced corals and thus, mitigate the deterioration of coral reef ecosystems.

## Materials and Methods

### *Exaiptasia pallida* Anemone Husbandry

Anemones used in these experiments were from the clonal CC7 population (John Pringle Lab, Stanford University) and were maintained in filtered artificial seawater at approximately 27 °C. Populations were kept on a day:night cycle of 12 h light:12 h dark with 30 to 60 μmole photons m^−2^ s^−1^ of light and fed freshly hatched brine shrimp nauplii twice a week. *E. pallida* used in this study were all approximately 3 mm in diameter and 10 mm high.

### Pathogenic Bacterial Species and Culture Preparation

*Vibrio coralliilyticus* strain BAA 450 (ATCC) was used for the infection experiment. To recover the bacteria from the glycerol stocks, the frozen culture was streaked out and grown overnight on Marine Agar (Difco, USA) at 30 °C. The following day a single colony was picked with an inoculating loop and grown to logarithmic phase at 30 °C in Marine Broth-2216 (Difco, USA) while shaking at 100 rpm. Cultures were centrifuged at 10,000 g for five minutes, washed and re-suspended in sterile seawater. Bacterial cells were prepared to the designated final concentration based on a growth curve of *V. coralliilyticus* generated with optical density readings at 600 nm plotted against known bacterial culture concentration (CFU).

### Determination of *Vibrio coralliilyticus* Concentration for Infection Trials

In order to determine an infective dose of the coral pathogen *Vibrio coralliilyticus*, ten-day infection trials were conducted on *E. pallida* anemones. Single anemones, acclimated to the experimental temperature (30 °C), were placed into single wells of a twelve-well culture dish containing filtered artificial seawater. During these trials, anemones (six per concentration) were challenged with three concentrations of the bacterium: 10^6^, 10^7^, or 10^8^ CFU ml^−1^ at 30 °C. *V. coralliilyticus* becomes virulent at temperatures greater than 28 °C^27^,^31^. The inoculation of bacteria was conducted using via balneation^56^. Anemones were monitored daily over ten days to assess behavioral changes and mortality events.

### Temperature Dependent Pathogen Virulence

To test if *V. coralliilyticus* showed similar temperature-dependent infectivity in *E. pallida* as it does in corals, infection trials were conducted at 25 °C and 30 °C for ten days. This experiment also allowed for the determination of the effect of experimental temperature on anemone survivorship. The concentration of *V. coralliilyticus* used for this experiment was 10^8^ CFU ml^−1^ as this inoculum dose showed the most consistent infectivity pattern on anemones (Supplementary Fig. S1A online). Four treatments were conducted (+Bacteria at 25 °C, +Bacteria at 30 °C, −Bacteria at 25 °C, and −Bacteria at 30 °C). Each treatment contained a total of six anemones. The anemones were allocated in single wells of twelve-well tissue culture plates. The inoculation of bacteria on the +Bacteria treated anemones were conducted using the balneation technique^56^. Anemones were monitored daily over ten days to assess behavioral changes and mortality events.

### Determination of Sub-lethal Pathogen Exposure

The sub-lethal exposure was not defined based on bacterial dose but based on the duration of exposure to the pathogen. From the infections experiments described above, the survivorship curves showed consistently that no anemone died during the first three days of pathogen exposure at a dose of 10^8^ CFU ml^−1^ (Supplementary Fig. S1 online). Based on these results, an additional experiment was conducted aiming to confirm if a 3-day exposure at 10^8^ CFU ml^−1^ to *V. coralliilyticus* could be considered a sub-lethal treatment. For this, anemones were exposed to *V. coralliilyticus* pathogen for a period of only three days, and placed in pathogen-free filtered seawater. The inoculation was carried out using via balneation^56^. The survivorship of these anemones was compared to a second group of anemones that remained under the bacterial exposure for a total of 10 days. Along with these two treatments, a control group of anemones were maintained in pathogen-free sea-water. The results from this experiment were fundamental to define the sub-lethal challenge used in subsequent priming experiments described below.

### Priming Experiments on *Exaiptasia pallida* anemones

All experiments in this section were conducted at 30 °C and at a final concentration of 10^8^ CFU ml^−1^ of *V. coralliilyticus*. Individual *E. pallida*, acclimated to the experimental temperature (30 °C) were placed into a single well of a twelve-well tissue culture plate containing filtered artificial seawater. Studies were conducted in three phases: sub-lethal exposure, recovery period, and lethal exposure. During the sub-lethal exposure, as indicated in the previous section, anemones (referred to as primed anemones) were challenged for three days with *V. coralliilyticus* (Supplementary Fig. S1B online). The sub-lethal exposures were followed by recovery periods, which varied as two, four, or six weeks. In this phase, anemones were removed from the sub-lethal exposure wells, transferred to pathogen-free seawater and left to recover during the designated recovery times. The final phase of the experiment consisted of the lethal exposure. From the previous experiment, it was determined that exposure to pathogen concentration of 10^8^ CFU ml^−1^ between 4 and 10 days was considered a lethal challenge (Supplementary Fig. S1 online). During this phase the primed anemones were challenged again with *V. coralliilyticus*. Additionally, a second treatment (defined as non-primed *E. pallida*) was composed of naive anemones that were not subjected to sub-lethal exposure but challenged with *V. coralliilyticus* in the last phase of the experiment. The control anemones were kept at 30 °C for the entire experiment but were never challenged with the bacterium.

### Quantitative PCR Assay for Determining *Vibrio coralliilyticus* Load on the Anemones

To determine the clearance dynamics of the *V. coralliilyticus* pathogen by the exposed anemones after the sub-lethal exposure time and during the lethal challenge, a quantitative real time PCR (qPCR) assay specific to test for pathogen presence was conducted. Anemone samples were collected immediately after finishing the sub-lethal exposure, and two and four days afterwards. During the lethal challenge (secondary exposure), anemones were collected at the following time points: one, three, seven and ten days after the start of the lethal exposure. For all the sampling times, three anemones were collected from each of the three treatments (primed, non-primed and controls). Total DNA was extracted from the collected anemones using the DNeasy Plant Mini Kit (Qiagen, Valencia, CA). DNA concentrations were estimated using the NanoDrop 2000c (Nano-Drop Technology, Wilmington, DE). *V. coralliilyticus* specific primers designed by Polson 2008 were used for the qPCR: 96F (5′-GTTRTCTGAACCTTCGGGGAACG-3′) and 1019R (5′- CTGTCTCCAGTCTCTTCTGAGG-3′)^72^. Reactions were conducted in 23 μl reactions with 12.5 μl of SYBR green master mix (BioRad, Hercules, CA) and 0.2 μM of each primer. The PCR conditions were as follows: 95 °C at 5 min followed by 40 cycles of 95 °C at 30 sec, 67 °C at 30 sec, and 72 °C at 60 sec. Dissociation curves were analyzed to confirm single product amplification at the end of qPCR runs. Samples were run in triplicate and mean values were used for calculations. Pathogen load on the infected anemones was expressed as relative proportion to amount of bacteria used at the initial inoculation (10^8^ CFU ml^−1^). For the calculations, we used the following equation; 2^(Ct^_i_^–Ct^_a_); where Ct_i_ represented the mean amplification cycle value for the DNA extracted from the initial inoculum (10^8^ CFU ml^−1^), and Ct_a_ represented the mean amplification cycle value for the DNA extracted from the infected anemone. A relative proportion of 1 means the pathogen load on an infected anemone corresponds to the same amount of pathogen found in concentration of 10^8^ CFU m^−1^.

### Statistical Analysis of Survivorship Data and Pathogen Load on Inoculated Anemones

For the priming experiments, Kaplan Meier estimators and survival plots were constructed for each of the three different recovery periods using the statistical software SPSS 21 (IBM). Post hoc comparisons using the Mantel–Cox test were further conducted to determine significant differences among the survival curves for each of the treatments. Analysis of variance (ANOVA) was performed in conjunction with a Tukey’s posthoc test to assess significance difference in the bacterial load on the anemones during different times after the sub-lethal challenge and during the lethal challenge using square root transformed data.

### Proteomic Analysis

In order to determine the molecular changes underpinning immunological priming in *E. pallida* anemones, a proteomic analysis was conducted by Applied Biomics (Hayward, CA) according to the company’s standard protocol. In this analysis, the proteomic profiles of anemones primed with the pathogen during a sub-lethal exposure were compared to naïve anemones never exposed to the pathogen four weeks after the experimental anemones were subjected to the sub-lethal exposure. Fifteen anemones were collected from each of two treatments. These fifteen anemones were pooled in three groups of five anemones each and snap frozen in liquid nitrogen. The pooling of anemones was necessary since it allowed enough tissue material for extraction of proteins to run the proteomic analyses. Samples were sent to Applied to Biomics (Hayward, CA) for two-dimensional differential in-gel electrophoresis (2D DIGE) profiling and separation. The 2D DIGE gels were scanned using a Typhoon image scanner (GE Healthcare). The images were analyzed using Image Quant software (GE-Healthcare), and then subjected to in-gel analysis and cross-gel analysis using DeCyder software version 6.5 (GE-Healthcare). Protein differential expression ratio changes were obtained by in-gel DeCyder software analysis. Of the spots that were differentially expressed, 32 were subjected to isolation using an Ettan Spot Picker (GE Healthcare) followed by MALDI-TOF (MS) using a 5800 mass spectrometer (AB Sciex).

Proteins were identified by submitting the peptide mass and fragmentation spectra to GPS Explorer version 3.5 using the MASCOT search engine (Matrix Science) where the National Center for Biotechnology Information non-redundant (NCBInr) database and *Exaiptasia pallida* genome (aiptasia.reefgenomics.org^73^) were explored. Significant candidates had either protein score C.I.% or Ion C.I.% greater than 95. Once the proteins were identified, gene ontology analysis was conducted using Blast2GO. E-values less than 1 × 10^−30^ and a percent identity of 97% were used as a cut off for identifying the gene fragments.

## Additional Information

**How to cite this article**: Brown, T. and Rodriguez-Lanetty, M. Defending against pathogens – immunological priming and its molecular basis in a sea anemone, cnidarian. *Sci. Rep.* **5**, 17425; doi: 10.1038/srep17425 (2015).

## Supplementary Material

Supplementary Information

## Figures and Tables

**Table 1:** 

2D Gel	Protein Identification	*Exaiptasia*	Protein	Protein	Fold	P
Spot		Genome	MW			value
Number		Gene ID	(Da)	PI	Change	T test
2	Heat Shock Protein (70 KDa)	AIPGENE12496	74,685	5.6	2.02	0.091
6	Signal Recognition Particle	AIPGENE24594	72,108	6.4	1.88	0.12
84	MRP Protein	AIPGENE1468	95,674	9.3	1.72	0.029
32	Calumenin-A	AIPGENE25880	35,247	4.5	1.69	0.015
34	Glutamate Receptor	AIPGENE24462	101,286	9.2	1.63	0.0081
50	Myosin Heavy Chain	AIPGENE8264	220,878	5.4	1.63	0.022
27	Aminopeptidase	AIPGENE26826	53,551	6.4	1.59	0.0046
3	Zona Pellucida	AIPGENE843	47,035	5.0	1.57	0.044
11	Heat Shock Protein (60 KDa)	AIPGENE15267	62,708	5.3	1.57	0.051
13	Moesin/ezrin/radixin	AIPGENE9804	66,884	5.8	1.56	0.064
43	Fructose-Bisphosphate Aldolase	AIPGENE2871	38,732	7.6	1.50	0.055
61	Heat Shock Protein (70 KDa)	AIPGENE12775	41,848	5.4	1.47	0.067
57	Voltage Dependent Anion Selective Channel	AIPGENE3808	35,188	9.1	1.45	0.027
82	Rho GDP-Dissociation Inhibitor	AIPGENE26434	22,251	4.8	1.32	0.12
65	Cathepsin	AIPGENE26157	36,124	6.7	1.25	0.041
42	Aspartate Aminotransferase	AIPGENE19338	45,969	6.8	−1.47	0.0043
87	Zinc Finger Protein	AIPGENE28354	68,856	8.8	−1.48	0.015
35	Cysteine Desulfurase	AIPGENE22361	54,104	6.2	−1.48	0.091
39	Fumarylacetoacetase	AIPGENE8362	46,370	6.2	−1.49	0.013
15	Selenium Binding Protein	AIPGENE13749	53,877	5.9	−1.50	0.0025
28	Cysteine Desulfurase	AIPGENE22361	54,104	6.2	−1.50	0.0046
14	Bleomycin Hydrolase	AIPGENE9335	55,335	5.8	−1.53	0.015
41	Pancreatic Triacylglycerol Lipase	AIPGENE19570	38,517	8.7	−1.55	0.025
71	Heat Shock Protein (70 KDa)	AIPGENE8252	41,986	5.3	−1.58	0.055
46	Calumenin B	AIPGENE9938	38,837	5.5	−1.59	0.0044
38	Cysteine Desulfurase	AIPGENE22361	54,104	6.2	−1.69	0.0021
56	Hemicentin	AIPGENE28714	49,371	6.6	−1.70	0.011
55	Thyroglobulin	AIPGENE20635	314,272	8.6	−1.85	0.0039
74	Nuclear Receptor	AIPGENE629	49,435	5.4	−1.91	0.046
70	Heat Shock Protein (70 KDa)	AIPGENE8252	41,986	5.3	−2.67	0.0018
68	Cyclic AMP and cGMP Phosphodiesterase	AIPGENE4644	51,903	5.8	−4.05	0.017
69	Heat Shock Protein (70 KDa)	AIPGENE8252	41,986	5.3	−9.73	0.0037

Identification of the 32 proteins subjected to Mass Spectrometry analysis. 2D Gel Spot Number: Number as indicated on the 2D-DIGE gel on supplemental Fig. 4; Protein Identification: Identity assigned by BLAST data base searches; Exaiptasia Genome Gene ID: Gene ID is based on the gene mapping identification for the *Exaiptasia* genome (aiptasia.reefgenomics.org^73^); Protein MW: Molecular weight of the protein; Protein PI: Isoelectric point of the protein; Fold Change: The fold change is based on protein expression of primed anemones versus non-primed (naïve) anemones; P Value T test: The significance probability value based on statistical T test.
