# Supplementary material for: Defending against pathogens – immunological priming and its molecular basis in a sea anemone, cnidarian
Source: Sci Rep. 2015 Dec 2;5:17425. doi: 10.1038/srep17425 (PMC4667181; doi:10.1038/srep17425)
Supplement: Supplementary Information [file srep17425-s1.pdf]

Supplementary Information

Title: "Defending against pathogens – immunological priming and its molecular basis in a sea anemone, cnidarian"

Authors:

Tanya Brown

Mauricio Rodriguez-Lanetty (*)

Address:

Department of Biological Sciences, Florida International University, Miami FL 33199

(*) Corresponding author

Keywords:

Invertebrate immunity, Immunological priming, coral immunology, Cnidarian, *Vibrio corallilyticus*

Supplemental Figure 1: Percent survival of *Exaiptasia pallida* to the exposure of the bacterial pathogen *Vibrio coralliilyticus* over ten days. A) Percent survival of *E. pallida* anemones under three different concentrations of *V. coralliilyticus* inoculum: 10^6, 10^7, and 10^8 CFU ml^{-1} at 30˚C. The inoculum concentration of 10^8 CFU ml^{-1} was chosen for the priming experiment since it showed the most consistent mortality results than using other inoculum concentration. B) Percent survival of *E. pallida* to the exposure of *Vibrio coralliilyticus* inoculum at different temperatures over ten days. Challenges were conducted at a concentration inoculum of 10^8 CFU ml^{-1} at both 25 and 30˚C. One hundred percent mortality was recorded at the bacterial challenge conducted at 30˚C by day seven of the experiment. Importantly, an increased temperature alone with no bacterial challenge did not cause mortality. Additionally, a bacterial challenge at 25˚C did not result in mortality over the ten-day experiment. These results indicated that bacterial infections of *Vibrio coralliilyticus* require to be performed at 30˚C, and that this experimental temperature has no negative effect on survivorship of *Exaiptasia pallida* anemones.

Supplemental Figure 2: Percent survival of *Exaiptasia pallida* anemones varying the exposure time to *V. coralliilyticus* at a dose of 10^8 CFU ml^{-1}: ten days, three days, and control. Results indicate that sea anemones show the same degree of survival to controls when only exposed to the bacterial pathogen, *V. coralliilyticus* for three days. The anemones that were exposed to the pathogen for ten days started dying after the fourth day of exposure. These results suggested that a 3-day exposure to the pathogen at $1x10^8$ CFU ml^{-1} is considered a sub-lethal treatment and was used as the priming condition in

the immunological experiments in this study. Exposure for more than four days is considered lethal.

Supplemental Figure 3: Relative Abundance of *Vibrio coralliilyticus* load in infected *Exaiptasia pallida* anemones estimated through specific quantitative PCR amplification of the pathogen 16S rDNA. A) Relative abundance of *V. coralliilyticus* load in infected anemones during the first four days after the completion of the sub-lethal exposure. The relative abundance of the pathogen is defined as the amount of bacterial load on the anemone in comparison to a bacterial concentration of $1x10^8$ CFU ml^{-1}. Day 0 represents anemones sampled immediately after being transferred to a new well for recovery at the completion of the sub-lethal exposure, Day 1 and Day 4 represent anemones sampled after one and four days following the completion of the sub-lethal exposure, respectively. Results indicate that *V. coralliilyticus* is cleared by *E.pallida* anemones by day four following the completion of the sub-lethal exposure (ANOVA, $p=0.04$, Tukey HSD, $p<0.05$). Error bars indicate standard deviation. B) Relative abundance of *V. coralliilyticus* load anemones during the ten-day lethal exposure. Day 1, 3, 7, and 10 represent days when anemones were sampled during the ten-day lethal exposure. Error bars indicate standard deviation. Results indicate that a considerable amount of *V. coralliilyticus* load is present in the tissue of *E. pallida* throughout the ten-day lethal exposure. The highest pathogen load was detected during the first three days of the challenge and then it declined at day 7 suggesting some level of clearance by the few anemones that had survived at this time (ANOVA, $p=0.01$; Tukey HSD, $p<0.05$).

Supplemental Figure 4: CyDye switch, two dimensional fluorescence difference gel electrophoresis (2D-DIGE) analysis of proteomes from naïve (N=3) and pathogen-primed (N=3) *Exaiptasia pallida* sea anemones. Naïve anemone samples were labeled with Cy3 (green) and primed anemone samples with Cy5 (red). Samples were then mixed and separated on analytical 2-D DIGE. The resulting gel was scanned and the merged image is shown where red proteins represent proteins whose expression is higher in the primed anemone tissue and green proteins represent proteins whose expression is higher in the naïve anemone tissue. The depicted gel is one example of the three replicated gels produced in this proteomic analysis. Circled and numbered spots represent proteins, which were most differentially expressed of which only those indicated by yellow circles were able to be analyzed using Mass-Spec and reported in Table 1. Red lines indicate protein numbers 2, 32, 46, 69, 70, and 71 pointing out the different isoforms detected of HSP70 and calumenin.

Supplemental Table 1: Blast2GO gene ontology terms provided for the 30 proteins analyzed by Mass Spectrometry.

A

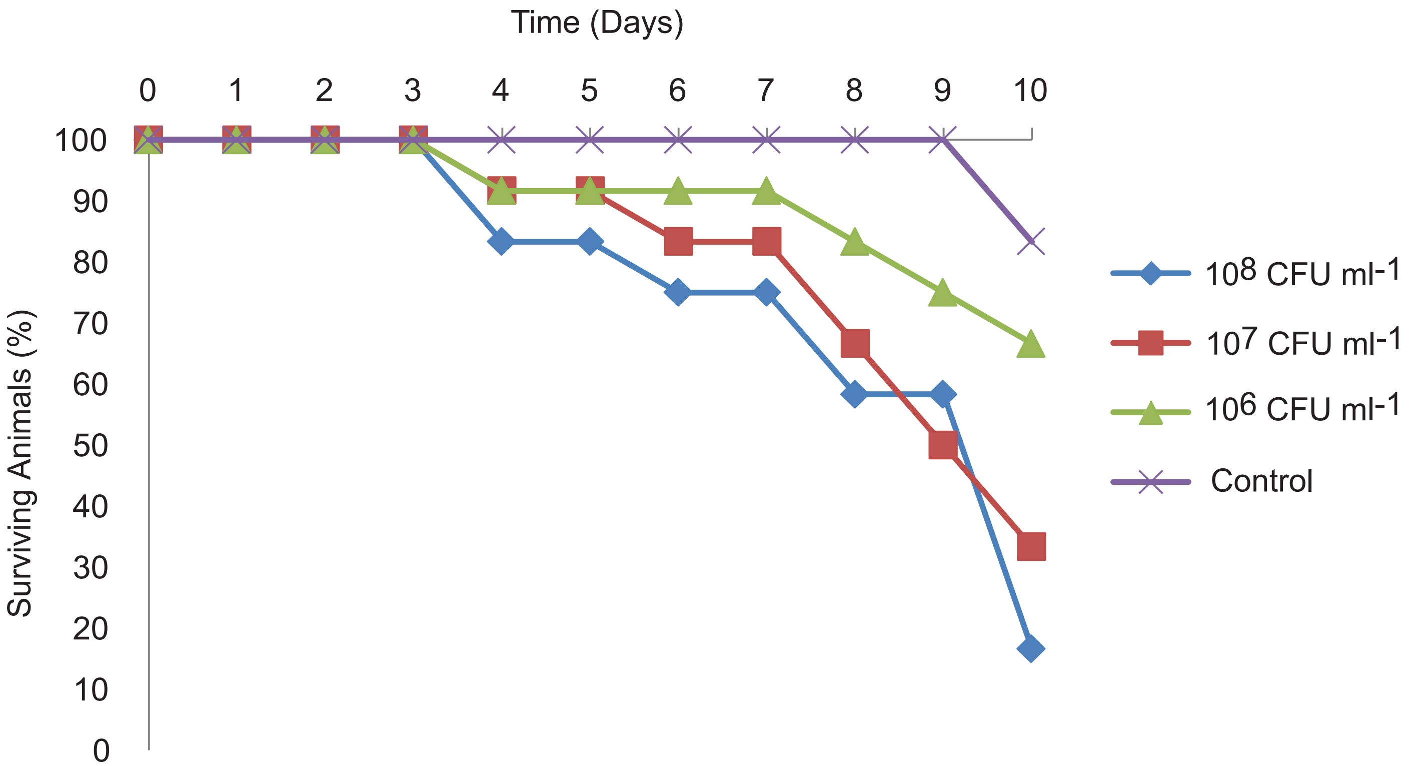

B

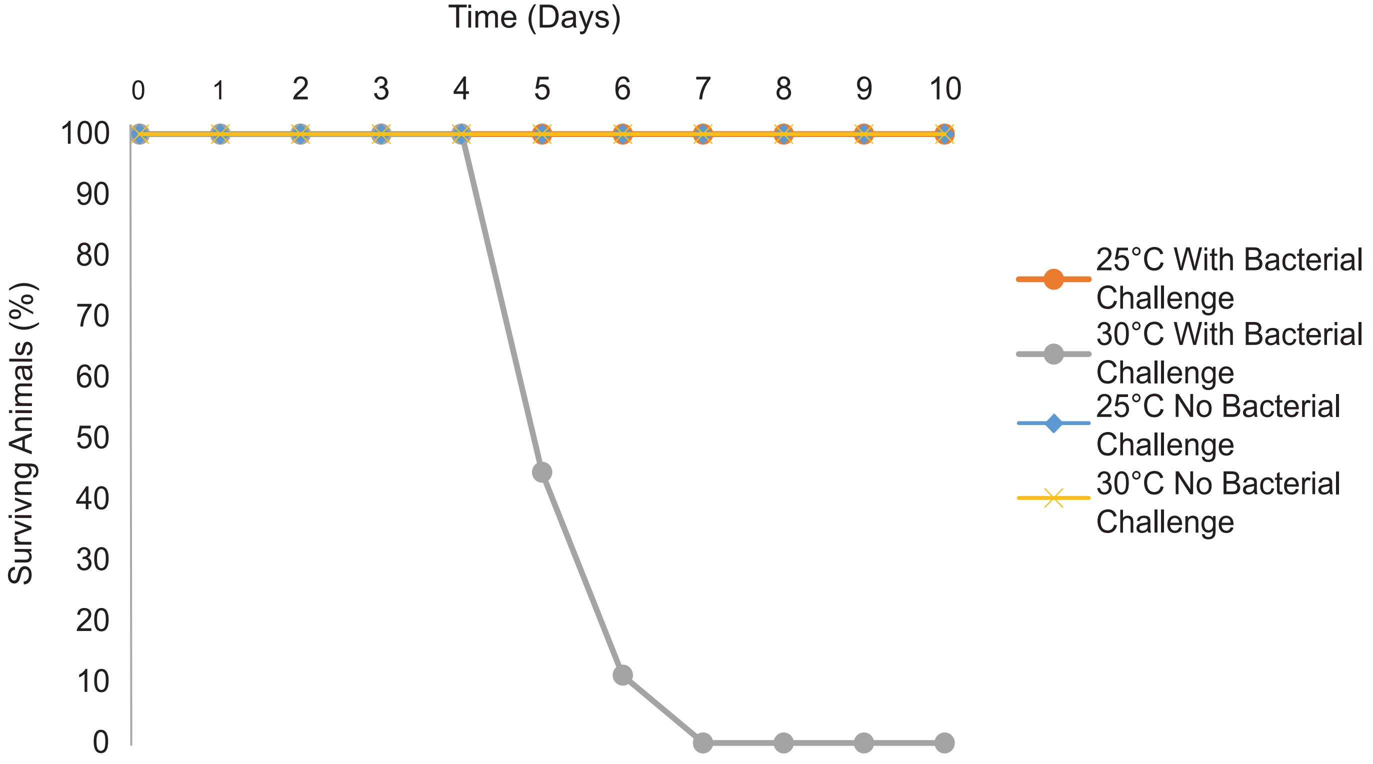

Supplemental S1

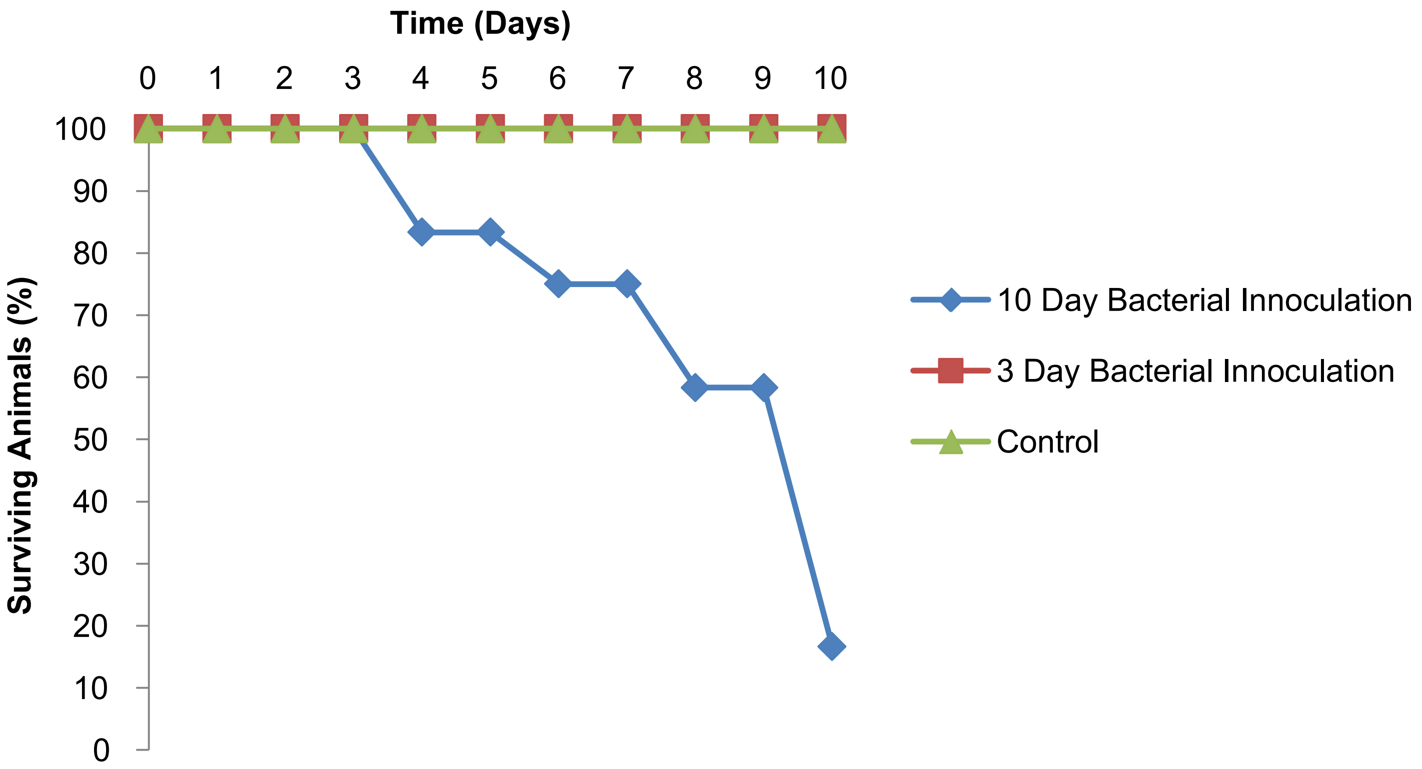

Supplemental S2

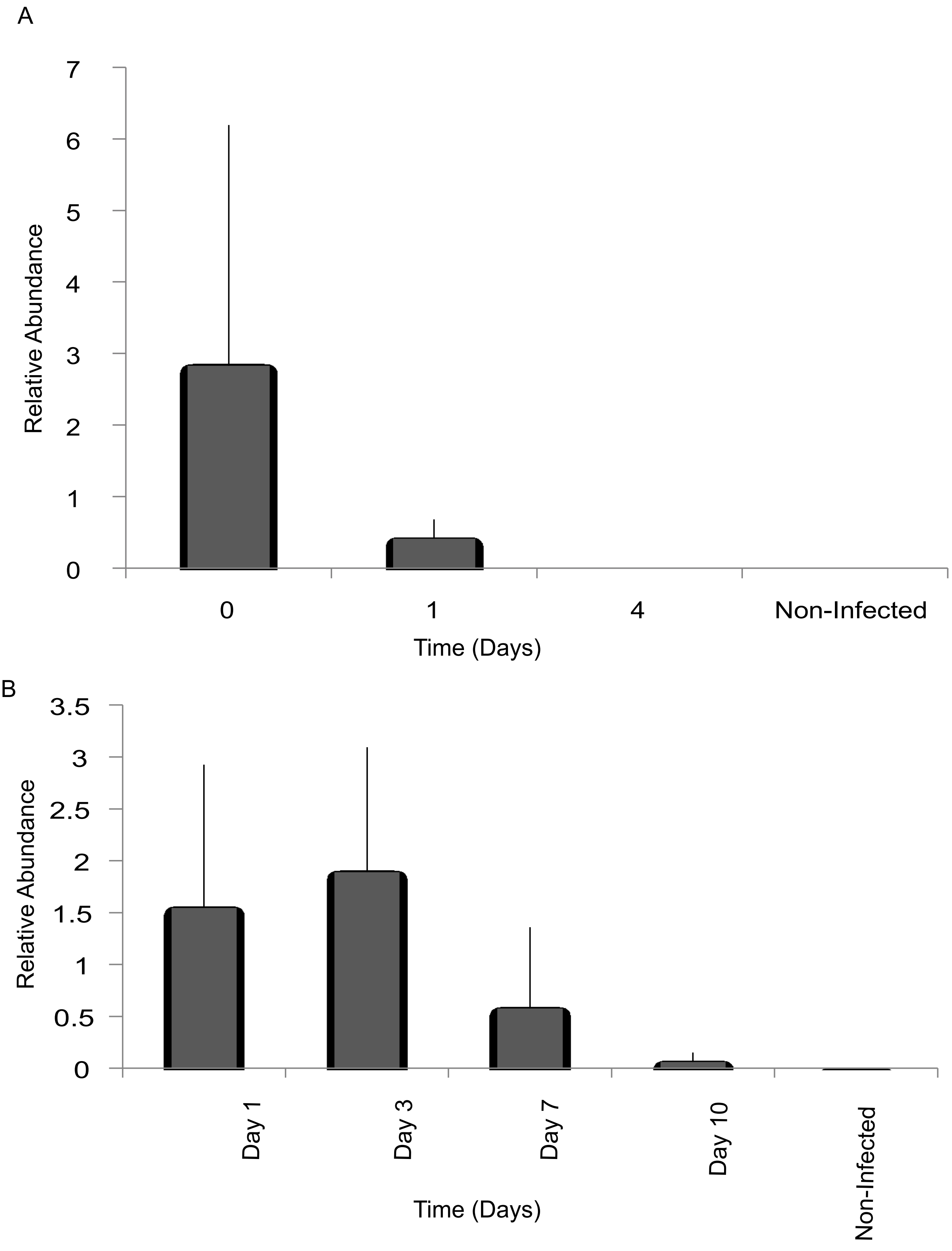

Supplemental S3

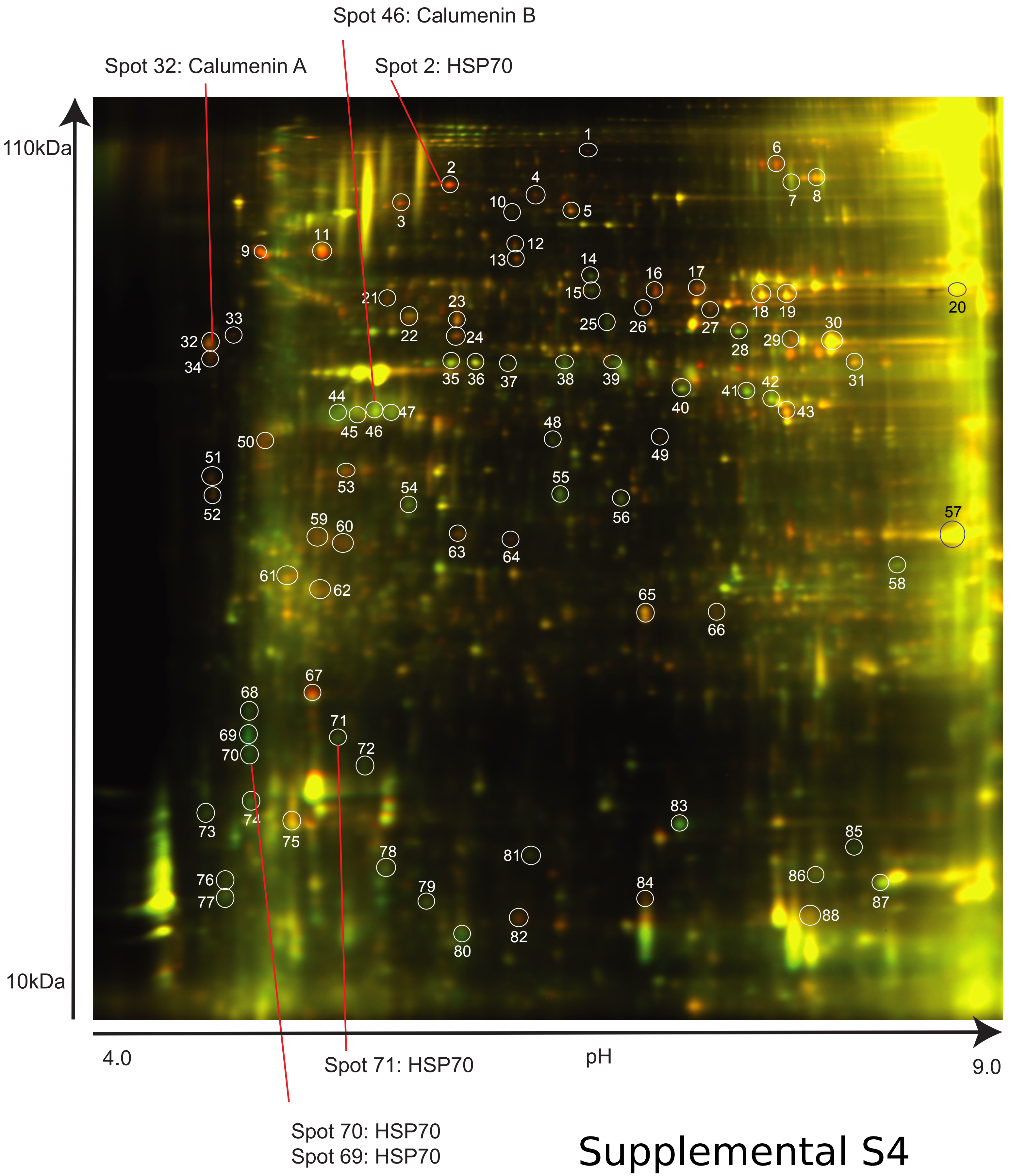

Supplemental S4

Supplemental Table 1

Spot Number	Protein ID	Aiptasia Gene ID	Protein MW	Protein PI	Fold Change	Biological process (GO Term)	Level 3 GO Terms
2	Heat Shock Protein (70 KDa)	AIPGENE 12496	74,685	5.6	2.02	Response to yeast, ER overload response, activation of signaling protein activity involved in unfolding protein response, cerebellum structural organization, cerebellar Purinje cell layer development, negative regulation of transforming growth factor beta receptor signaling pathway, positive regulation of protein ubiquination, cellular response to glucose starvation, negative regulation of apoptotic process, proteolysis involved in cellular protein catabolic process, cellular response to interleukin -4	Response to stress, cellular response to stimulus, single-organism cellular process, anatomical structure development, single multicellular organism process, single organism developmental process, regulation of biological process, response to endogenous stimulus, response to chemical, catabolic process, organic substance metabolic substance cellular response to stimulus
6	Signal Recognition Particle	AIPGENE 24594	72,108	6.44	1.88	GTP catabolic process, SRP-dependent	Single organism cellular process

						cotranslational protein targeting to membrane	
84	MRP Protein	AIPGENE 1468	95,674	9.3	1.72	Metabolic process	Single-organism metabolic process, nitrogen compound metabolic process, cellular metabolic process, organic substance metabolic process
32	Calumenin - A	AIPGENE 25880	35,247	4.5	1.69	proteolysis	Primary metabolic process, organic substance metabolic process
34	Glutamate Receptor	AIPGENE 24462	101,28 6	9.2	1.63	Ion transmembrane transport, ionotropic glutamate receptor signaling pathway	Single organism cellular process, single organism localization, single organism signaling, cellular response to stimulus, regulation of biological process
50	Myosin Heavy Chain	AIPGENE 8264	220,87 8	5.4	1.63	NA	
27	Aminopeptida se	AIPGENE 26826	53,551	6.4	1.59	Proteolysis, regulation of catalytic activity, protein metabolic process	Primary metabolic process, organic substance metabolic process, regulation of molecular function
3	Zona Pellucida	AIPGENE 843	47,035	5.0	1.57	Receptor mediated endocytosis, cell adhesion, calcium ion binding, negative regulation of peptidase activity	Establishment of localization, organic substance metabolic process, regulation of molecular function
11	Heat Shock Protein (60 KDa)	AIPGENE 15267	62,708	5.3	1.57	Fin regeneration, protein refolding, oxaloacetate metabolic process,	Single organism metabolic process, organic substance metabolic process, cellular metabolic process, primary

						glycerol biosynthetic process, aspartate biosynthetic process, aspartate catabolic process, response to hormone, glutamate catabolic process to aspartate, glutamate catabolic process t 2-oxoglutarate, fatty acid homeostasis	metabolic process, nitrogen compound metabolic process, response to chemical, single organism cellular process, regulation of biological quality
13	Moesin/Ezrin/ Radixin	AIPGENE 9804	66,884	5.8	1.56	Positive regulation of gene expression, cellular component organization, establishment of endothelial barrier	Organic substance metabolic process, regulation of biological process, cellular component organization, single organism developmental process, single organism cellular process, anatomical structure development
43	Fructose-Bisphosphate Aldolase	AIPGENE 2871	38,732	7.6	1.5	Glycolytic process	Cellular metabolic process, nitrogen compound metabolic process, organic substance metabolic process, primary metabolic process, single-organism metabolic process, catabolic process
61	Heat Shock Protein (70 KDa)	AIPGENE 12775	41,848	5.4	1.47	NA	
57	Voltage Dependent Anion	AIPGENE 3808	35,188	9.1	1.45	Anion transport, fin regeneration, regulation of anion	Single organism localization, establishment of localization, response to stress, single

	Selective Channel					transport, transmembrane transport	organism developmental process, anatomical structure development, establishment of localization, regulation of biological process
82	Rho GDP-Dissociation Inhibitor	AIPGENE 26434	22,251	4.8	1.32	Proteolysis, regulation of catalytic activity, protein metabolic process	Organic substance metabolic process, primary metabolic process, regulation of molecular function
65	Cathepsin	AIPGENE 26157	36,124	6.7	1.25	Proteolysis, regulation of catalytic activity, protein metabolic process	Organic substance metabolic process, primary metabolic process, regulation of molecular function
42	Aspartate Aminotransfer ase	AIPGENE 19338	45,969	6.8	-1.47	Oxaloacetate metabolic process, glycerol biosynthetic process, aspartate biosynthetic process, aspartate catabolic process, response to hormone, glutamate catabolic process to aspartate, glutamate catabolic process to 2-oxoglutarate, fatty acid homeostasis	Single organism metabolic process, organic substance metabolic process, cellular metabolic process, single organism cellular process, biosynthetic process, primary metabolic process, nitrogen compound metabolic process, response to chemical, regulation of biological quality
87	Zinc Finger Protein	AIPGENE 28354	68,856	8.78	-1.48	Regulation of transcription, DNA template, chitin metabolic process	Organic substance metabolic process, nitrogen compound metabolic process, cellular metabolic process, biosynthetic process
35	Cysteine Desulfurase	AIPGENE 22361	54,104	6.2	-1.48	Metabolic process	Nitrogen compound metabolic process, primary metabolic

							process, cellular metabolic process, organic substance metabolic process
39	Fumarylacetoacetase	AIPGENE 8362	46,370.7	6.2	-1.49	Arginine catabolic process, aromatic amino acid family metabolic process	Nitrogen compound metabolic process, organic substance metabolic process, cellular metabolic process, single organism cellular process, single organism metabolic process
15	Selenium Binding Protein	AIPGENE 13749	53,877	5.9	-1.5	Protein transport, brown fat differentiation	Single organism developmental process, single organism cellular process
28	Cysteine Desulfurase	AIPGENE 22361	54,104	6.2	-1.5	Metabolic process	Nitrogen compound metabolic process, primary metabolic process, cellular metabolic process, organic substance metabolic process
14	Bleomycin Hydrolase	AIPGENE 9335	55,335	5.8	-1.53	Proteolysis, response to drug	Organic substance metabolic process, primary metabolic process, response to chemical
41	Pancreatic Triacylglycerol Lipase	AIPGENE 19570	38,517	8.7	-1.55	Lipid metabolic process	Organic substance metabolic process, primary metabolic process, single organism metabolic process
71	Heat Shock Protein (70 KDa)	AIPGENE 8252	41,986	5.3	-1.58	NA	
46	Calumenin B	AIPGENE 9938	38,837	5.5	-1.59	NA	
38	Cysteine Desulfurase	AIPGENE 22361	54,104	6.2	-1.69	Metabolic process	Nitrogen compound metabolic process, primary metabolic process, cellular metabolic

							process, organic substance metabolic process
56	Hemicentin	AIPGENE 28714	49,371	6.6	-1.7	Chitin metabolic process	Organic substance metabolic process, nitrogen compound metabolic process
55	Thyroglobulin	AIPGENE 20635	314,27 2.6	8.56	-1.85	Cell matrix adhesion, negative regulation of endopeptidase activity, negative regulation of peptidase activity	Organic substance metabolic process, regulation of biological process, regulation of molecular function
74	Nuclear Receptor	AIPGENE 629	49,435. 8	5.39	-1.91	Regulation of transcription, intracellular receptor signaling pathway, response to lipid, organ development	Single organism signaling, cellular response to stimulus, single organism cellular process, regulation of biological process, response to chemical, single multicellular organism process, single organism developmental process
70	Heat Shock Protein (70 KDa)	AIPGENE 8252	41,986	5.3	-2.67	NA	
68	Cyclic AMP and cGMP Phosphodieste rase	AIPGENE 4644	51,903	5.8	-4.05	NA	
69	Heat Shock Protein (70 KDa)	AIPGENE 8252	41,986	5.3	-9.73	NA	
